# A DNA-centered explanation of the DNA polymerase translocation mechanism

**DOI:** 10.1038/s41598-017-08038-2

**Published:** 2017-08-08

**Authors:** J. Ricardo Arias-Gonzalez

**Affiliations:** 1 Instituto Madrileño de Estudios Avanzados en Nanociencia, C/Faraday 9, Cantoblanco, 28049 Madrid, Spain; 2 CNB-CSIC-IMDEA Nanociencia Associated Unit “Unidad de Nanobiotecnología”, Madrid, Spain

## Abstract

DNA polymerase couples chemical energy to translocation along a DNA template with a specific directionality while it replicates genetic information. According to single-molecule manipulation experiments, the polymerase-DNA complex can work against loads greater than 50 pN. It is not known, on the one hand, how chemical energy is transduced into mechanical motion, accounting for such large forces on sub-nanometer steps, and, on the other hand, how energy consumption in fidelity maintenance integrates in this non-equilibrium cycle. Here, we propose a translocation mechanism that points to the flexibility of the DNA, including its overstretching transition, as the principal responsible for the DNA polymerase ratcheting motion. By using thermodynamic analyses, we then find that an external load hardly affects the fidelity of the copying process and, consequently, that translocation and fidelity maintenance are loosely coupled processes. The proposed translocation mechanism is compatible with single-molecule experiments, structural data and stereochemical details of the DNA-protein complex that is formed during replication, and may be extended to RNA transcription.

## Introduction

A polymerase is a motor protein that transfers genetic information inside biological cells. There exist two types of polymerases, DNA and RNA polymerase (DNAp and RNAp)^1, 2^. The former replicates a DNA template strand into a complementary DNA strand, a process known as replication that is needed for cell division. The latter transfers the information of a DNA template into an RNA transcript in the so-called transcription process. The product of replication is a double-helix DNA molecule made up of two complementary strands and that of transcription is a double-helix hybrid molecule made up of a DNA strand and a complementary RNA strand. DNAp and RNAp translocate on a stepwise fashion on consecutive DNA template positions, hydrolyzing triphosphate into monophosphate nucleosides for their ultimate incorporation into the replicate or transcript strand. Although statistical and kinetic models have described the stochastic behavior and information processing by DNAp and RNAp^3–8^, it is not known how the chemical free energy obtained from this hydrolysis reaction is transduced into the polymerase mechanical motion.

In contrast, motion and force generation mechanism are clear in other motor proteins. Kinesin hydrolyzes ATP into ADP plus phosphate (Pi). ATP binding to the forward head attaches this head to the microtubule track and ATP hydrolysis in the rear head releases this head from the microtubule. After ATP hydrolysis, the protein can move either forward or backward by ratcheting and, on average, it performs a net forward step by bringing the rear head to the front by using the asymmetric torsional strain accumulated between its two tails in the helical stalk. Rotational steps of ATPases^9, 10^ or bacteriophage packaging mechanisms and force generation, see refs 11 and 12 for reviews, have also been analyzed. Kinesin withstands maximum forces of ≈7 *pN* under saturating ATP conditions^13^, a load limit that is related to the torsional strain that can be accumulated in the helical stalk, and moves in 8-*nm* steps, a length that is related, on the one hand, to the average separation between heads after a 180-degree rotation of one head over the other in its hand-over-hand movement and, on the other hand, to the tubulin-dimer length.

**Figure 1:**
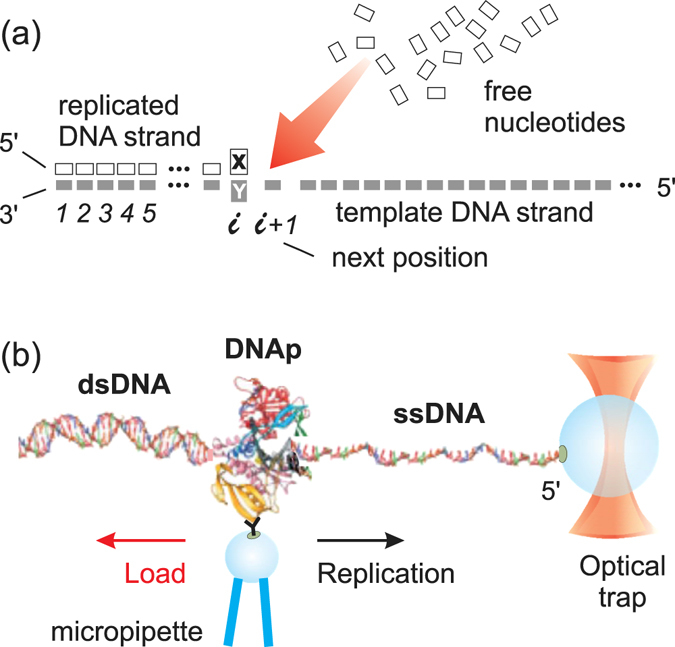
DNA replication scheme. (**a**) Abstract view of the single nucleotide addition reaction. DNA polymerase replicates a template strand from its 3′-end to its 5′-end by incorporating nucleotides on a one-by-one basis and translocating to adjacent positions *i* → *i* + 1. (**b**) Simplified configuration for measuring load-dependent dynamics in single-molecule experiments (not to scale). A DNA polymerase (DNAp) chemically bound to a bead on a micropipette replicates a single-stranded DNA (ssDNA), which 5′ end is chemically bound to an optically-trapped bead, and generates double-stranded DNA (dsDNA). The optical trap is the force sensor.

DNAp translocates stepwisely on a DNA polymer^14^, as depicted in Fig. 1, which is a flexible structure. It moreover produces strain on this molecular track, unlike kinesin, whose microtubular track can be considered rigid with respect to the strength of the forces developed by this transport motor. The flexibility of double-stranded DNA (dsDNA) has been thoroughly characterized in single-molecule experiments (see ref. 15 for a review). Two almost linear elasticity regimes and a transition to an almost unwound state have been described for this polymer. At low forces ($\mathop{ < }\limits_{ \tilde {}}$5 *pN*), the dsDNA aligns straight with the applied force; at higher forces, ≈5 − 65 *pN*, the polymer is stretched intrinsically. This regime of elasticity, which is thus known as *intrinsic* or *enthalpic*, can be characterized by a Young modulus, like a macroscopic material. At ≈65 *pN* the copolymer experiences an overstretching transition to 1.7 times its contour length.

In the following, we explain how the flexibility of the DNA double-helix polymer that results from replication is involved in the mechanism by which DNAp moves with a specific directionality and withstands high forces. We then analyze the consequences for the maintenance of fidelity. Our analysis allows the thermodynamic efficiency of these information biomachines to be understood.

## Analysis

### The stacking of the nascent base-pair triggers the translocation step of the DNA polymerase

The precise structure of the overstretched double-stranded nucleic acids involves a process of unstacking of the base-pairs and an almost total unwinding of the double helix structure, which may be accompanied by DNA melting, depending on conditions^15^. The biological relevance of the overstretching transition in dsDNA has not been clearly determined because no DNA binding motor protein that can work against such a high force has been measured to date. Viewed from the opposite side, an overstretched dsDNA accumulates a high spring-like potential energy. In particular, a single base-pair (bp) that relaxes to a stacked, double-stranded conformation releases an energy

1 $${\rm{\Delta }}{g}_{os}={F}_{os}\times {\rm{\Delta }}l,$$

being *F*_*os*_ the overstretching force and Δ*l* the distance difference between two consecutive base-pairs in the overstretched state with respect to the double-helix state. Let *l* be the distance between base-pairs in the double-helix polymer; then, according to single-molecule force-extension measurements^15^, the following heuristic relation holds:

2 $$\frac{{\rm{\Delta }}l}{l}\approx 0.7,$$

being *l* ≈ 0.34 *nm*/*bp* in dsDNA (B form)^16, 17^. This relation must be considered on a sequence-average basis since it actually depends on the nucleotides involved in consecutive base-pairs and conditions^18, 19^.

The structure of a DNAp resembles a right hand with the template DNA wired through the palm domain and the thumb and fingers folding around it. The fingers domain fluctuates between an open and close conformation so that the DNAp can grab deoxyribonucleoside triphosphates (dNTPs) from the environment on a one-by-one basis and test them on the corresponding template nucleotide. In this state, the DNAp sustains the template strand sharply bent to nearly 90 degrees inside the enzyme structure^20–26^. A dNTP that is selected as a correct one, according to Watson-Crick complementarity, is docked probably helped by a spontaneous H-bonding process with concomitant stabilization of the DNAp closed conformation^27^. This dNTP-bound conformation also consolidates the post-translocated state of the enzyme with respect to its previous position on the template strand^28^. DNAp then catalyzes the phosphodiester bond formation to attach the newly incorporated deoxyribonucleoside monophosphate (dNMP) to the previous one on the replicated strand. The energy for this process is obtained from the hydrolysis of the dNTP into dNMP + pyrophosphate (PPi).

Then, we propose the following *DNA*-*centered translocation mechanism* in DNA replication: Soon after the new nucleotide is branched to the replicated strand, the newly formed base-pair experiences a (majorly) hydrophobic interaction with the previous base-pair, thus triggering a conformational change. This conformational change involves a sudden stacking of the two initially distant base-pairs hence forming the double-stranded arrangement, possibly by combining a 33–36 degrees twist inherent to the double helical conformation^17, 18^. In these conditions, the newly formed double-helix fragment slides within the DNAp structure thus sitting the so-called ‘pol’ site of the protein into the next position in the template strand.

**Figure 2:**
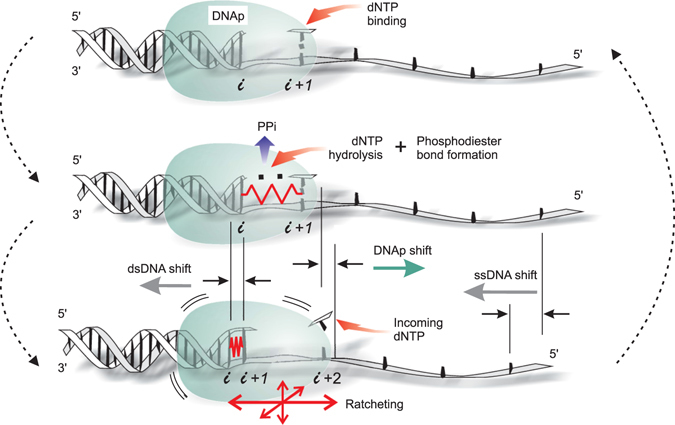
Role of DNA in the translocation mechanism of DNA polymerase. From top to bottom and back to top (follow dashed arrows), the diagram shows the single-nucleotide addition reaction in DNA replication with emphasis on how chemical energy is transduced into mechanical work (approximately to scale). Top panel, a suitable dNTP (normally keeping Watson-Crick complementarity) H-bonds to the template dNMP at position *i* + 1. Middle panel, this dNTP is hydrolyzed by a phosphoryl transfer reaction. As a result, a phosphodiester bond is formed with the previously replicated dNMP and a pyrophosphate (PPi) is released. Due to the distance between the nucleotides in the single-stranded template, the newly formed base-pair emerges overstretched, as represented by the red spring. This triggers a sudden stacking into the previous base-pair at *i* and shifts DNA and DNA polymerase (DNAp) in opposite directions, hence giving rise to a net translocation into position *i* + 2. The bottom panel closes the cycle: The DNAp can now thermally diffuse like a Brownian ratchet until another dNTP docks, which stabilizes the post-translocated state. If this new dNTP is suitable, the cycle restarts (top panel); otherwise, it is released until a different dNTP arrives. Mechanical stress of the DNAp, elastic conformational changes of the DNAp and bending of the DNA by the DNAp have not been represented for the sake of simplicity.

The mechano-chemical cycle of DNAp is represented in Fig. 2 according to the above ideas. As explained, our model focuses on the role of the DNA flexibility in the translocation step of the DNAp, considering the elasticity of the enzyme to have a secondary role^3^. The herein proposed mechanism, in contrast to the kinesin case in which the microtubule remains rigid, points at the molecular track as the responsible of DNAp translocation. The asymmetry of the described mechanism, in which the new base-pair stacks from the 3′ end to the 5′ end of the replicated strand, marks the directionality of the polymeration process. Next, we analyze the maximum load that the DNAp can withstand under this mechanism, showing coherence with single-molecule experiments^28^.

### The mechanical work of an external load is mainly buffered by the DNA flexibility

The energy released upon incorporation of the newly formed base pair onto the previous one can be assumed as a relaxation of the new base-pair from an overstretched to a stacked arrangement. Then, the maximum force exerted by a physiological load on the DNA-DNAp complex is limited by the overstretching transition of the double-stranded polymer. At physiological conditions (pH ~ 7.5, 100 *mM* monovalent salt concentration, NaCl), this force is ≈65 *pN*. This transition is highly cooperative for dsDNA: it expands over less than 2 *pN*. However, it is important to note that DNAp locally dehydrates the DNA due to binding^29, 30^. This effect may lower the overstretching force down to ~40 *pN* and expand the transition over as much as 30 *pN* for very low salt concentration.

In single-molecule experiments, this load is acted by a device (normally in an optical tweezers system, see Fig. 1b), which opposes to the non-equilibrium successive linear translocation steps $i\to i+1\to i+2\ldots$ on the template strand. According to our DNA-centered model, the associated work, *W*, is mainly absorbed by the DNA flexibility, see Fig. 2. It can be therefore expressed as *W* = −*F*Δ*x*, where the force and distance fulfill *F* ≤ *F*_*os*_ and Δ*x* ≤ Δ*l* below the overstretching transition, and the sign of the force indicates that it is against translocation. For an overstretching force of *F*_*os*_ ≈ 65 *pN* and a relaxation of the newly formed base-pair from the overstretched to the B-form distance Δ*l* ≈ 0.34 × 0.7 ≈ 0.24 *nm*, it is clear that Δ*g*_*os*_ ≈ 3.8 *kT*, with *k*, the Boltzmann constant and *T*, the temperature (equations (1) and (2)).

The energy dissipated in the mechanical translocation, *ε*_*m*_, appears from the release of the newly formed base-pair into a stacked and winded conformation in the growing dsDNA. According to our model, it can be estimated from

3 $${\varepsilon }_{m}={\rm{\Delta }}{g}_{os}-{\rm{\Delta }}{g}_{stack},$$

where Δ*g*_*stack*_ is the stacking free energy of the newly formed base-pair, which we consider positive for Watson-Crick unions (opposite convention as that used in ref. 31). In general, the energy excess *ε*_*m*_ also depends on the structural strain in the complex between the DNAp and the DNA template. The associated elastic energy is therefore DNAp-dependent.

The DNA accumulates elastic energy along the enthalpic elasticity regime in the sugar-phosphate backbone. During the overstretching transition —which is, according to our model, the inverse process—the high tension disrupts the base-stacking and the helical conformation and, depending on conditions, this process may be accompanied by DNA melting. Therefore, Δ*g*_*os*_ > Δ*g*_*stack*_.

*ε*_*m*_ may slightly differ from equation (3) but it comes, in any case, from a separate energy slot of that used for fidelity according to our model, as we analyze in greater depth next.

### Fidelity maintenance consumes a substantial amount of the nucleotide hydrolysis energy

The cartoon of Fig. 2 does not account for the effect of a mismatch in the structure of the resulting dsDNA nor does it represent the mechanism by which non-equilibrium energy is consumed to preserve the template information.

It is known that conformational changes oriented to regulate fidelity allow the detection of correct H-bonding/fraying of the incoming nucleotide and, more stringently, the geometry recognition of the resulting base-pair in the binding pocket based on size and shape and on solvent exclusion^25, 26, 32^. The resulting structural fitting of the DNAp to the DNA substrate, not only to the nascent base-pair but also to previous neighbors (memory^33^), has been explained to amplify the stacking free energies of the base-pairs^31^ yielding much lower error rates (enthalpy-entropy compensation^34^) than those resulting from the equilibrium process with a passive DNAp^6^.

These considerations have been addressed in recent kinetic formalisms of growth velocity and fidelity during the real, non-equilibrium process, these models including nearest neighbor and higher-order effects^35–38^. In addition, within the context of information theory and thermodynamics of DNA replication, it has been recently shown that to keep the fidelity within a given tolerance, average consumed energy *E* must increase with the reliability of the incorporated nucleotide according the next relation^33^ (see Methods):

4 $${p}_{error}(E)\approx \,\mathrm{ln}\,({e}^{\beta E}+3{e}^{-\beta E})-\beta E\frac{{e}^{\beta E}-3{e}^{-\beta E}}{{e}^{\beta E}+3{e}^{-\beta E}},$$

where *p*_*error*_ is the sequence-independent probability of error per incorporated nucleotide (or error rate) in the absence of exonucleolytic proofreading and *β* = 1/*kT*. This approximated expression becomes valid for $\beta E\mathop{ > }\limits_{ \tilde {}}2$ ^33^. Being only thermodynamic, this model is of predictive value for the relationship between energetics and fidelity, but it is not for the relation between fidelity and growth rates.

The single-nucleotide addition in replication can be represented by the following oversimplified reaction (see also Figs 1a and 2):

5 $$DN{A}_{i}+dNTP\leftrightarrow DN{A}_{i+1}+PPi,$$

where DNA stands for the replicated strand. In the case of transcription, the released standard free energy, which we take for estimate purposes, is ${\rm{\Delta }}{g}_{i\to i+1}^{0}=2.1\pm 0.8\,kcal/mol$ (absolute value)^39^. For physiological conditions, namely, *T* ≈ 298 − 310 *K* and [*dNTP*] ≈ 1 *mM*, [*PPi*] ≈ 0.001 *mM*, this energy is of Δ*g*_*i*→*i*+1_ ≈ 6.2 *kcal*/*mol* or approximately 10 *kT*. This energy comprises the hydrolysis of the dNTP (negative) and the (positive) energy invested in the phosphodiester bond formation of the resulting dNMP at position *i* + 1.

Considering the energies from the above coupled reactions, the energy balance for the translocation step implies:

6 $$E={\rm{\Delta }}{g}_{i\to i+1}+{\rm{\Delta }}{g}_{os}-{\varepsilon }_{m}-{\varepsilon }_{f}+W,$$

where *ε*_*f*_ (positive) accounts for the energy dissipated in fidelity maintenance. We consider positive both the heat absorbed by the system and the work supplied to the system, which is the DNA-DNAp complex.

Equation (6) indicates that a mechanical stress counteracts the available free energy thus decreasing fidelity in the presence of dissipation. For external forces above the entropic regime of elasticity of the DNA, $F\mathop{ > }\limits_{ \tilde {}}5\,pN$, the tension over this molecule further stretches the DNA structure, Fig. 1b. The Worm-like chain model can be used to account for the change in the distance between base-pairs in the enthalpic elasticity regime^40, 41^,

7 $$\frac{{\rm{\Delta }}x}{l}=-\frac{1}{2}\,{(\frac{kT}{F{L}_{p}})}^{\mathrm{1/2}}+\frac{F}{S},$$

where *L*_p_ and *S* are the persistence length and stretch modulus, respectively, of the polymer. Although this expression is also valid in the interphase between the entropic and enthalpic regimes, it cannot be herein used when Δ*x* < 0. Such a negative change is mainly related to a decrease in the end-to-end distance of the DNA, which stochastically folds under the action of both its electric charge in a polar medium and the thermal fluctuations^15, 41^. It is thus not strictly related to an intrinsic change in contour length, which would appear as a consequence of a decrease in the distance between base-pairs. Since the nascent base-pair is constrained within the DNAp structure, the work done by the external load does not affect the DNA-DNAp complex (*W* = 0) until the enthalpic elasticity regime, in which it opposes the stacking of this nascent base-pair. Then, when the external load is used to align the DNA, this work is not related to the nucleotide incorporation process.

It must be stressed here that the entropic and enthalpic DNA elasticity regimes may exhibit some overlapping for Δ*x* < 0 in equation (7), i.e. that partial base-pair unstacking and entropic alignment of the chain may co-exist at low forces. However, the change in rise per base-pair at near entropic forces would make $W\mathop{ > }\limits_{ \tilde {}}0$.

**Figure 3:**
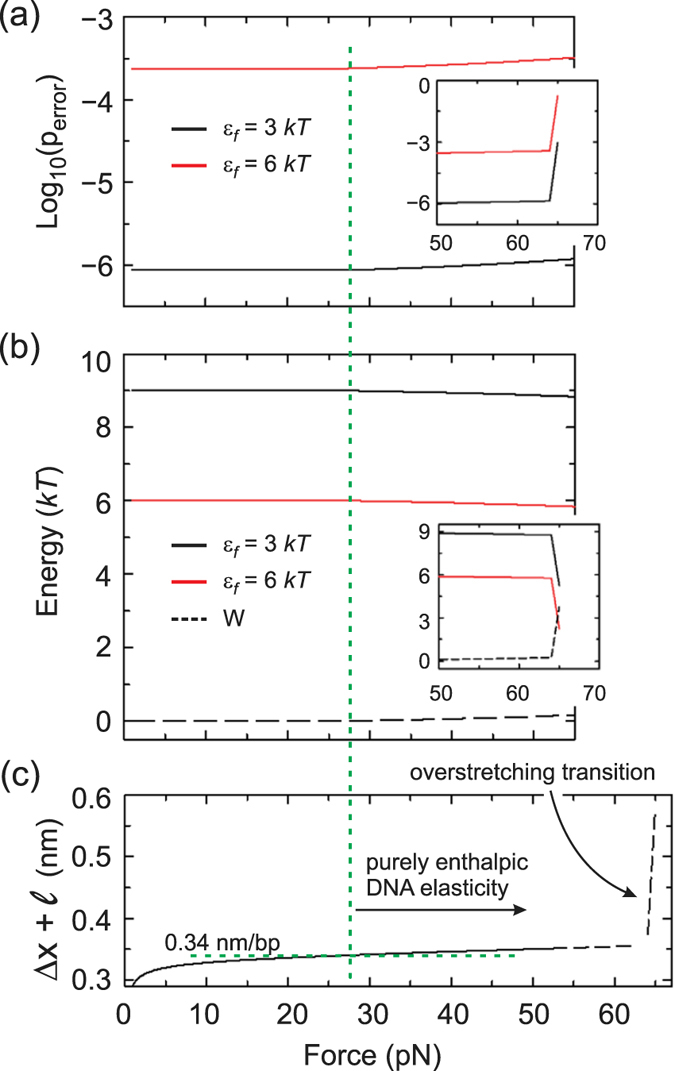
Balance between translocation work and fidelity maintenance in the absence of proofreading. Error rate in logarithmic scale, (**a**), and energy invested in fidelity, (**b**), are plotted as functions of an external load. The insets show these plots near and at the overstretching transition. Two fidelity dissipation energies have considered, *ε*_*f*_ = 3 and 6 *kT*, black and red traces, respectively (see the text for details). Black, dashed curve in (b), work done by the external load. (**c**) Distance between base-pairs as a function of an external load. The DNA parameters are *l* = 0.34 *nm*, *L*_*p*_ = 50 *nm* and *S* = 1000 *pN* and the free energies Δ*g*_*i*→*i*+1_ = 10 *kT*, Δ*g*_*os*_ = 4 *kT* and Δ*g*_*stack*_ = 2 *kT* (*ε*_*m*_ = 2 *kT*). Green, dashed horizontal line marks the distance between base-pairs in B-DNA. The vertical green, dashed line accross panels demarks the DNA purely enthalpic elasticity regime.

The energetics of our model are plotted in Fig. 3 as a function of an external force. As shown in panel (a), the error rate increases in the presence of mechanical stress but it hardly affects its order of magnitude. This is due to the fact that, as proposed, the work of the external force is majorly consumed in stretching the nascent DNA base-pair, thus leaving the energy coming from the dNTP hydrolysis (after phosphodiester bond formation) for fidelity maintenace, Fig. 3b. The insets in (a) and (b) show the hypothetical case in which the external force were as large as the overstretching transition (see panel (c)). In that case, the energy left for fidelity maintenace would be very low and the error rate would dramatically increase. Activities at forces above 50 *pN* have been detected with the Φ29 DNAp^28^. The extrapolation of the velocity-force curves in that report, however, indicates that the activities slow down to zero before the overstretching transition.

In these conditions, our model shows that the energy used for fidelity is almost preserved since translocation is majorly associated to the energy coming from the stacking of the nascent base-pairs near overstretching distances. Our model reflects that fidelity of DNA polymerases is not abruptly changed in the presence of mechanical stress in the cell and that the mechanism of translocation is loosely coupled to that oriented to balance fidelity.

### Thermodynamic efficiency

Assuming the energy consumed in translocation and in fidelity maintenance, the efficiency of the DNAp can be estimated as

8 $$\eta =\frac{E-W}{{\rm{\Delta }}{g}_{i\to i+1}+{\rm{\Delta }}{g}_{os}}=1-\frac{{\varepsilon }_{m}+{\varepsilon }_{f}}{{\rm{\Delta }}{g}_{i\to i+1}+{\rm{\Delta }}{g}_{os}},$$

where *E* is a free parameter that contains the stacking free energies of the base-pairs in general conditions, including those of dehydration generated by the DNAp during its activity, and *F* (in *W*) is another free parameter that addresses the DNA elasticity regimes, both according to the fidelities and translocation mechanism described above. In this regard, to adjust the model to experimental error rates measured on typical replicative DNA polymerases, we have considered two fidelity dissipation energies in Fig. 3: *ε*_*f*_ = 3 and 6 *kT*. The former gives rise to an error rate of ~10^−6^ and the latter, to ~10^−4^ (Fig. 3a) in the absence of proofreading. These data yield respective efficiencies of *η* ≈ 64% and 43%. The error rate of the second case is similar to that of Φ29 DNAp^42, 43^. If we consider that this DNAp is able to perform strand displacement activity as a helicase and consider two extra 2 *kT* of useful work in the thermodynamic efficiency^7^, this yields *η* = 57%, more similar to the other case.

These efficiencies are consistent with those of transport biomachines like the kinesin^13^. This protein consumes 1 ATP molecule (≈20 *kT*) and generates a mechanical work of *W* ≈ 7 *pN* × 8 *nm* ≈ 13 *kT*. Then, *η* ≈ 65%. The fact that information consumes energy provides a more realistic efficiency for DNAp than the apparent low efficiency calculated only based in the mechanical translocation activity^44^.

## Discussion

We have performed a thermodynamic analysis of the single-nucleotide addition cycle in DNA replication, the first that conjugates mechanical and information aspects. We propose that the DNA elasticity has a central role in the translocation of the DNAp relative to the DNA substrate. This includes the overstretchig transition, which associated DNA structure change at high force are observed as the reverse of the base-pairing and base-stacking processes that take place during nucleotide incorporation in DNA replication.

Our model is consistent with a ratchet mechanism in which there is a loose coupling between chemical catalysis and mechanical translocation during DNA replication^28^ and explains that DNA replication attains directionality by the way the base-pairs stack on each other. Base-pair stacking then provides an asymmetry in the potential barrier that the DNAp overcomes after burning one dNTP to rectify thermal fluctuations. According to our model, the translocation of the DNAp is only limited by external forces near the DNA overstretching transition because the work done by the force is mostly absorbed by the DNA flexibility. High opposing loads below the overstretching transition force do not abruptly change the DNA rise per base-pair and thus the base-stacking process of the nascent base-pairs. These forces, then, while hindering and slowing the DNAp, do not necessarily stall the protein activity, as experimentaly found^28^. This makes reasonable that translocation rates are approximately load-independent for low forces directly applied to DNA substrate^45, 46^.

The integration of information theory results^33^ allows us to conclude that the mechanical work used for translocation is almost independent of the energy spent in maintaining fidelity in the replication process. Based on our model and previous biochemical, structural and single-molecule data, fidelity controls can be re-arranged into four checkpoints along the mechano-chemical cycle of the DNAp: The first corresponds to the dNTP binding stage (Fig. 2, from bottom to top panel). The second implies a conformational transition in the ternary complex to accomodate the dNTP before it is hydrolyzed (Fig. 2, top panel). The third, slow, after the phosphoryl transfer reaction of this dNTP but before PPi release, corresponds to a second conformational change of the complex after the chemistry (Fig. 2, middle panel)^47, 48^. And the fourth one, also slow, after PPi release, is associated with the Brownian ratcheting of the whole DNAp around the recently reached position, after the new base-pair has stacked (position *i* + 2, Fig. 2, bottom panel)^28, 45, 49^. The structural fitting of the DNAp to both the nascent and previous DNA base-pairs (memory)^6, 25, 33, 49^ affects the DNAp stepping dynamics. Conformational changes are slower and more inefficient for the extension of a mismatch than for a correct nucleotide^25, 26, 32, 47, 48^ making the protein *toddle* rather than *walk* relative to the DNA, a dynamics which enables error detection and subsequent exonucleolytic proofreading.

We indeed find that fidelity maintenance consumes the major part of the free energy available at each replication step making the overall thermodynamic efficiency for the DNAp information ratchet consistent with that found for kinesin, a transport motor protein. It is then expected that the principal conformational changes of DNA polymerases, more than towards achieving net steps with respect to the DNA template, are oriented to fidelity maintenance. These changes are, besides, protein-dependent, as needed to account for the diverse fidelities and structural details of the different polymerases.

## Methods

In the following, we derive the error rate in the absence of proofreading, equation 4. The entropy for replication in the absence of exonucleolytic proofreading, *S* ^(*D*)^, according to Eq. (32) in ref. 33, reads:

9 $${S}^{(D)}(\beta E)=k\,\,\mathrm{ln}\,{Z}^{(id)}(\beta E)-kn\beta E{{\rm{\Lambda }}}^{(id)}(\beta E),$$

where we have used that the number of characters in the genetic alphabet is 4 (i.e., ${\mathscr{X}}=\{A,C,G,T\}$, cardinality $|{\mathscr{X}}|=4$), that *n* is the number of nucleotides in the chain and functions *Z* ^(*id*)^ and Λ^(*id*)^ are given by Eqs (25) and (34) in the same reference, namely:

10 $${Z}^{(id)}(\beta E)={({e}^{\beta E}+3{e}^{-\beta E})}^{n},$$

11 $${{\rm{\Lambda }}}^{(id)}(\beta E)=\frac{{e}^{\beta E}-3{e}^{-\beta E}}{{e}^{\beta E}+3{e}^{-\beta E}}.$$

where, again, we have used that the genetic alphabet is made up of 4 elements.

Appendix D in that paper explains how to obtain the error rate from the Shannon-McMillan-Breiman theorem (or general *Asymptotic Equipartition Theorem*) and the concept of *typical set* ^50^. Then, from Eq. (D4) in ref. 33

12 $${p}_{error}\to 1-\exp \,(-\frac{{s}^{(D)}}{k})\simeq \frac{{s}^{(D)}}{k},$$

where *S* ^(*D*)^ is the entropy per nucleotide, namely, *s* ^(*D*)^ = *S* ^(*D*)^/*n*, and the approximation becomes good for low *S* ^(*D*)^. Now, inserting equation (9) in (12) for *s* ^(*D*)^ = *S* ^(*D*)^/*n* we find equation (4). The approximation $\exp \,(-{s}^{(D)}/k)\simeq {s}^{(D)}/k$ becomes valid for $\beta E\mathop{ > }\limits_{ \tilde {}}2$ ^33^, which is the energetic level of the stacking energies in DNA^31^.

Note that, from a mathematical viewpoint, equation (4) can also be interpreted as a probability in the limit *E* → 0. Certainly, equation (4) without the approximation yields *p*_*error*_(*E* = 0) = 3/4, which corresponds to the case in which the four nucleotides are equally probable: since only one is valid, the probability of correct incorporation is 1/4 and that of error is 3/4.

A corresponding error rate can be obtained for fidelity in the presence of exonucleolytic proofreading by using the entropy *S* of Eq. (31) in ref. 33 and following the same steps. Since this process involves a different reaction with corresponding energy source, we do not use it here to evaluate the efficiency of the DNAp step during nucleotide incorporation.
